# A consensus linkage map for molecular markers and Quantitative Trait Loci associated with economically important traits in melon (*Cucumis melo *L.)

**DOI:** 10.1186/1471-2229-11-111

**Published:** 2011-07-28

**Authors:** Aurora Diaz, Mohamed Fergany, Gelsomina Formisano, Peio Ziarsolo, José Blanca, Zhanjun Fei, Jack E Staub, Juan E Zalapa, Hugo E Cuevas, Gayle Dace, Marc Oliver, Nathalie Boissot, Catherine Dogimont, Michel Pitrat, René Hofstede, Paul van Koert, Rotem Harel-Beja, Galil Tzuri, Vitaly Portnoy, Shahar Cohen, Arthur Schaffer, Nurit Katzir, Yong Xu, Haiying Zhang, Nobuko Fukino, Satoru Matsumoto, Jordi Garcia-Mas, Antonio J Monforte

**Affiliations:** 1 Instituto de Biología Molecular y Celular de Plantas (IBMCP). Universidad Politécnica de Valencia (UPV)-Consejo Superior de Investigaciones Científicas (CSIC). Ciudad Politécnica de la Innovación (CPI), Ed. 8E. C/Ingeniero Fausto Elio s/n, 46022 Valencia, Spain; 2 IRTA, Center for Research in Agricultural Genomics (CSIC-IRTA-UAB), Campus UAB, Edifici CRAG, 08193 Bellaterra (Barcelona), Spain; 3 Department of Soil, Plant, Environmental and Animal Production Sciences, Federico II University of Naples, Via Università 100, 80055 Portici, Italy; 4 COMAV-UPV, Institute for the Conservation and Breeding of Agricultural Biodiversity, Universidad Politécnica de Valencia, Camino de Vera s/n, 46022 Valencia, Spain; 5 Boyce Thompson Institute for Plant Research, Ithaca, New York 14853, USA; 6 USDA-ARS, Vegetable Crops Research Unit, Department of Horticulture, 1575 Linden Dr, University of Wisconsin, Madison, WI 53706, USA; 7 Current address: USDA-ARS, Forage and Range Research Laboratory, Utah State University, Logan, UT 84322-6300, USA; 8 Current address: USDA-ARS, Tropical Agricultural Research Station, 2200 Pedro Albizu Campus Ave, Mayaguez 00680-5470, Puerto Rico; 9 Syngenta Biotechnology, Inc. Research Triangle Park, NC 27709, USA; 10 Syngenta Seeds, 12 chemin de l'Hobit, F-31790 Saint-Sauveur, France; 11 INRA, UR 1052, Unité de Génétique et d'Amélioration des Fruits et Légumes, Domaine St Maurice, BP 94, 84143 Montfavet Cedex, France; 12 Keygene N.V. P.O. Box 216. 6700 AE Wageningen. The Netherlands; 13 Institute of Plant Science, Agricultural Research Organization (ARO), Newe Ya'ar Research Center, Ramat Yishay 30095, Israel; 14 Institute of Plant Science, Agricultural Research Organization, Volcani Research Center, Bet Dagan 50250, Israel; 15 National Engineering Research Center for Vegetables (NERCV), Beijing Academy Agricultural and Forestry Science, Beijing 100097, China; 16 National Institute of Vegetable and Tea Science (NIVTS), 360 Kusawa, Ano, Tsu, Mie, 514-2392, Japan; 17 Agronomy Department Faculty of Agriculture, Ain Shams University, Cairo, Egypt

## Abstract

**Background:**

A number of molecular marker linkage maps have been developed for melon (*Cucumis melo L*.) over the last two decades. However, these maps were constructed using different marker sets, thus, making comparative analysis among maps difficult. In order to solve this problem, a consensus genetic map in melon was constructed using primarily highly transferable anchor markers that have broad potential use for mapping, synteny, and comparative quantitative trait loci (QTL) analysis, increasing breeding effectiveness and efficiency via marker-assisted selection (MAS).

**Results:**

Under the framework of the International Cucurbit Genomics Initiative (ICuGI, http://www.icugi.org), an integrated genetic map has been constructed by merging data from eight independent mapping experiments using a genetically diverse array of parental lines. The consensus map spans 1150 cM across the 12 melon linkage groups and is composed of 1592 markers (640 SSRs, 330 SNPs, 252 AFLPs, 239 RFLPs, 89 RAPDs, 15 IMAs, 16 indels and 11 morphological traits) with a mean marker density of 0.72 cM/marker. One hundred and ninety-six of these markers (157 SSRs, 32 SNPs, 6 indels and 1 RAPD) were newly developed, mapped or provided by industry representatives as released markers, including 27 SNPs and 5 indels from genes involved in the organic acid metabolism and transport, and 58 EST-SSRs. Additionally, 85 of 822 SSR markers contributed by Syngenta Seeds were included in the integrated map. In addition, 370 QTL controlling 62 traits from 18 previously reported mapping experiments using genetically diverse parental genotypes were also integrated into the consensus map. Some QTL associated with economically important traits detected in separate studies mapped to similar genomic positions. For example, independently identified QTL controlling fruit shape were mapped on similar genomic positions, suggesting that such QTL are possibly responsible for the phenotypic variability observed for this trait in a broad array of melon germplasm.

**Conclusions:**

Even though relatively unsaturated genetic maps in a diverse set of melon market types have been published, the integrated saturated map presented herein should be considered the initial reference map for melon. Most of the mapped markers contained in the reference map are polymorphic in diverse collection of germplasm, and thus are potentially transferrable to a broad array of genetic experimentation (e.g., integration of physical and genetic maps, colinearity analysis, map-based gene cloning, epistasis dissection, and marker-assisted selection).

## Background

Saturated genetic linkage maps (< 1 cM between markers) are required for the efficient and effective deployment of markers in plant breeding and genomic analysis. Linkage map applications include, but are not limited to: gene mapping, positional cloning, QTL analysis, MAS, epistasis dissection, linkage disequilibrium analysis, comparative genomics, physical and genetic map integration, and genome assembly. The construction of highly saturated maps is often a time-consuming process, especially if investigators are employing different parental stocks and markers are not easily transferable. Merged maps are attractive since their integration allows for an increase in marker density without the need of additional genotyping, increased marker portability (i.e., polymorphic markers can be used in more than one population), improved marker alignment precision (i.e., congruent anchor maker position), and broader inferential capabilities (i.e., cross-population prognostication). A number of integrated linkage maps have been developed in numerous economically important crop plants including grapevine (*Vitis vinifera *L.) [1], lettuce (*Lactuca sativa *L.) [2], maize (*Zea mays *L.) [3], red clover (*Trifolium pratense *L.) [4], ryegrass (*Lolium ssp.*) [5], wheat (*Triticum aestivum *L.) [6], among others.

The genome of melon (*Cucumis melo *L.; 2n = 2x = 24) is relatively small (450 Mb, [7]), consisting of 12 chromosomes. The first molecular marker-based melon map was constructed in 1996 [8] using mainly restriction fragment length polymorphism (RFLP) markers and morphological traits, although the markers did not cover the predicted 12 melon chromosomes. This was comparatively late for a major crop species like melon that is among the most important horticultural crops in terms of worldwide production (25 millions of tons in 2009) and which production has been increased around 40% in the last ten years [9]. Subsequently, the first linkage maps that positioned markers on 12 linkage groups (LG) were constructed few years later, using the F_2 _progeny of a cross between the Korean accession PI161375 and the melon type "Pinyonet Piel de Sapo" [10] and two Recombinant Inbred Line (RIL) populations derived from the crosses "Védrantais" × PI161375 and "Védrantais" × PI414723 [11]. However, these maps had few markers in common and different LG nomenclature, making comparative mapping intractable. More recently, dense linkage maps have been constructed using Simple Sequence Repeat (SSR) [12-16] and Single Nucleotide Polymorphism (SNP) [17,18] markers. Nevertheless, although these maps share common markers, they possess large numbers of map-specific markers that makes map-wide comparisons complicated.

Melon germplasm displays an impressive variability for fruit traits and response to diseases [19-22]. Recently, part of this variability has been genetically dissected by QTL analysis [18,23-27]. Inter-population QTL comparisons among these maps are, however, difficult given the aforementioned technical barriers.

Databases integrating genomic, genetic, and phenotypic information have been well developed in some plant species such as the Genome Database for Rosaceae [28], SOL Genomics Network for Solanaceae [29] or Gramene [30], and provide powerful tools for genomic analysis. In 2005, the International Cucurbit Genomics Initiative (ICuGI) [31] was created to further genomic research in Cucurbitaceae species by integrating genomic information in a database (http://www.icugi.org). Thirteen private seed companies funded this project, which sought to construct an integrated genetic melon map through merging existing maps using common SSR markers as anchor points. We present herein an integrated melon map, including the position of QTL controlling economically important traits, to facilitate comparative mapping comparison and to create a dynamic genetic backbone for the placement of additional markers and QTL.

## Results and discussion

### Construction of the integrated map

#### Anchor molecular markers

Based on their previously observed even map distribution, polymorphism, and repeatability, 116 SSR markers and 1 SNP marker (Additional File 1) were chosen as anchor points to integrate the eight genetic maps (Table 1). Anchor marker segregation varied among maps, where the greatest number of polymorphic anchor markers were in IRTA (Institut de Recerca i Tecnologia Agroalimentáries, Barcelona, Spain) [15] and INRA (Institut National de la Recherche Agronomique, Montfavet Cedex, France) [11] maps containing 100 and 82 anchor polymorphic markers, respectively. The minimum number of anchor polymorphic markers was recorded in the NERCV (National Engineering Research Center for Vegetables, Beijing, China) [32] map (35 polymorphic markers). Most of the anchor markers were originally mapped in the IRTA population, that shared a common parent (the Korean line PI 161375) with the INRA population, while the other parent was an Occidental cultivar ("Piel de Sapo" and "Vedrantais" for IRTA and INRA populations, respectively), so it was actually expected that the proportion of markers that can be transferred successfully from IRTA to INRA populations is larger than to the any other studied population developed from different germplasm.

**Table 1:** Mapping populations

Map	Parental lines	Subspecies	Market class	Horticultural group	Population type	Population size	Number of markers	Number of polymorphic anchor markers	Maximum number of shared markers	Map length (cM)	Reference
INRA	Védrantais	*melo*	Charentais	*cantalupensis*	RIL	154	223	82	68	1654	[11,27]
	PI 161375	*agrestis*		*chinensis*							

ARO	Dulce	*melo*	Cantaloup	*reticulatus*	RIL	94	713	56	64	1222	[18]
	PI 414723	*agrestis*		*momordica*							

IRTA	Piel de sapo	*melo*	Piel de sapo	*inodurus*	DHL	69	238	100	111	1244	[15]
					DHL	14	528				[17]
	PI 161375	*agrestis*		*chinensis*	F2	93	293	37	111	1197	[10]

NITVS	AR 5	*melo*	Cantaloup	*reticulatus*	RIL	93	228	70	70	877	[16]
	Hakurei 3	*melo*	Cantaloup	*reticulatus*							

NERCV	K7-1	*melo*	Hami melon	*cantalupensis*	RIL	107	237	35	41		[32]
	K-7-2	*melo*	Hami melon	*cantalupensis*							

USDA	USDA 846-1	hybrid			RIL	81	245	37	64	1116	[13]
	Top Mark	*melo*	Western	*reticulatus*							
			Shipper								
	Top Mark	*melo*	Western	*reticulatus*							
	Q 3-2-2	*melo*	Shipper	*conomon/*	F2	117	168	35	64	1095	[14]
				*momordica*							
											

Summary of the mapping populations used to construct the integrated map. Each map is named by the abbreviation of the collaborating institutions (INRA, Institut National de la Recherche Agronomique, France; ARO, Agricultural Research Organization, Israel; IRTA, Institut de Recerca i Tecnologia Agroalimentàries, Spain; NITVS, National Institute of Vegetable and Tea Science, Japan; NERCV, National Engineering Research Center for Vegetables, China; and USDA-ARS U. S. Department of Agriculture, Agricultural Research Service, USA). The genotypes used as mapping parents belong to the subspecies (*Cucumis melo *L.: ssp. *melo *or *C. melo *ssp. *agrestis*), and the market class and horticultural group are classified according to Pitrat et al. (2000) [49]. The DHL population of 14 genotypes is actually a selected sample for bin mapping of the 69 DHLs [12]. The number of polymorphic anchor markers segregating within each map and the maximum number of markers shared by each map with at least one of the other maps are also shown.

#### Molecular marker segregation analysis among individual maps

Considerable and significant skewed marker segregations (p < 0.005) were detected in seven genomic regions of the DHL-based IRTA map (Table 1). Although significant skewed segregations were also detected in a region on LG VIII of the F2-based IRTA map [10], on LGs I, IV, and VI in NIVTS (National Institute of Vegetable and Tea Science, Mie, Japan) map [116] and on LGs V, VII, VIII and X in the ARO (Agricultural Research Organization, Ramat Yishay 30095, Israel) map [18]. No significant segregation distortion was detected in the other maps used herein (data not shown). The relatively high number of genomic regions with skewed segregation detected in the DHL-based map reinforces the hypothesis that such distortion likely originated from unintentional selection during the *in vitro *line development process [33]. The low number of genomic regions showing skewed segregation in most melon maps contrasts with that reported in other crops such as lettuce [2], red clover [4], sorghum [34], and tomato [35]. The degree of such distortion has been correlated to the extent of taxonomic divergence between mapping parents [36]. The use of inter-specific hybrids in order to construct genetic maps is a common strategy to ensure the availability of a high number of polymorphic markers, and in such cases segregation distortion may be frequent [37]. However, depending on the relative frequency and intensity, segregation distortion may not interfere on the map construction. Nevertheless, such distortion may hinder the transfer of economically important alleles during plant improvement. The comparatively low frequency of segregation distortion present in melon maps may be partially explained by the use of intra-specific crosses during population development. Given the infrequent occurrence of segregation distortion in melon, the introgression of novel, economically important alleles from exotic melon germplasm into elite modern cultivars should be relatively unimpeded.

#### Marker polymorphism and recombination rates among individual maps

The number of polymorphic markers for individual maps ranged from 168 (USDA-ARS, Vegetable Crops Research Unit, Department of Horticulture, Madison USA) to 713 (ARO) (Table 1). INRA and IRTA maps consisted of 12 LGs, coinciding with the basic chromosome number of melon, whereas the remaining maps consisted of more LGs (see http://www.icugi.org for further details). The number of common markers in pairwise individual map comparisons was quite variable, with a mean of 40 common markers among maps. Each individual map shared between 41 and 111 markers with at least one of the other maps (Table 1). Marker order and recombination rates among markers were very consistent among maps, where significant recombination rate heterogeneities (p < 0.001) were detected between only a few marker pairs (CMN22_85-CMTCN66 in LGIII, CMAGN75-CMGA15 in LG VII, and TJ2-TJ3 in LG VIII). Similar results have been found during genetic map integration in grapevine [1], but more frequent recombination rate differences have been reported among integrated maps in apple (*Malus domestica *Borkh) [38], *Brassic*a ssp. [39], and lettuce [2]. Differences in locus order and recombination rates may be attributed, in part, to bands that were scored as single alleles instead of duplicated loci or to evolutionary events (chromosomal rearrangements). Nevertheless, it must be concluded from the data presented that major chromosomal rearrangements have not occurred during the recent evolutionary history (i.e., domestication) of this species.

#### Consensus linkage map

The construction of the integrated map described herein involved two stages: 1) the building of a framework map by merging all the available maps (Table 1) using Joinmap 3.0 [40]; 2) the addition of subsequent markers using a "bin-mapping" approach [41].

Given the high co-linearity among melon maps, 1565 markers from all maps were initially employed for map integration. However, 258 (16%) of these markers could not be included in the final integrated map. This proportion was smaller than that obtained during map integration of lettuce (19.6% [2]), and larger than in the grapevine integrated map (8%, [1]). The markers segregating within each individual map were quite complementary, what made the inclusion of a large number of markers into the final merged map possible. For example, the IRTA_F2 map was constructed with an important proportion of RFLP markers that were not used in most of the other maps. However, this map had enough RFLP markers in common with the IRTA_LDH map, which has a good proportion of common markers with INRA (68) and NIVTS (70) maps, making possible to integrate the IRTA_F2 RFLP markers in the final map.

Given the congruency detected among melon maps, the inability to incorporate some previously mapped markers into the integrated map is likely due to the lack of sufficient linkage among markers in some genomic regions, especially in small LGs drawn from some individual maps where there was a paucity of common framework map markers.

**Figure 1:**
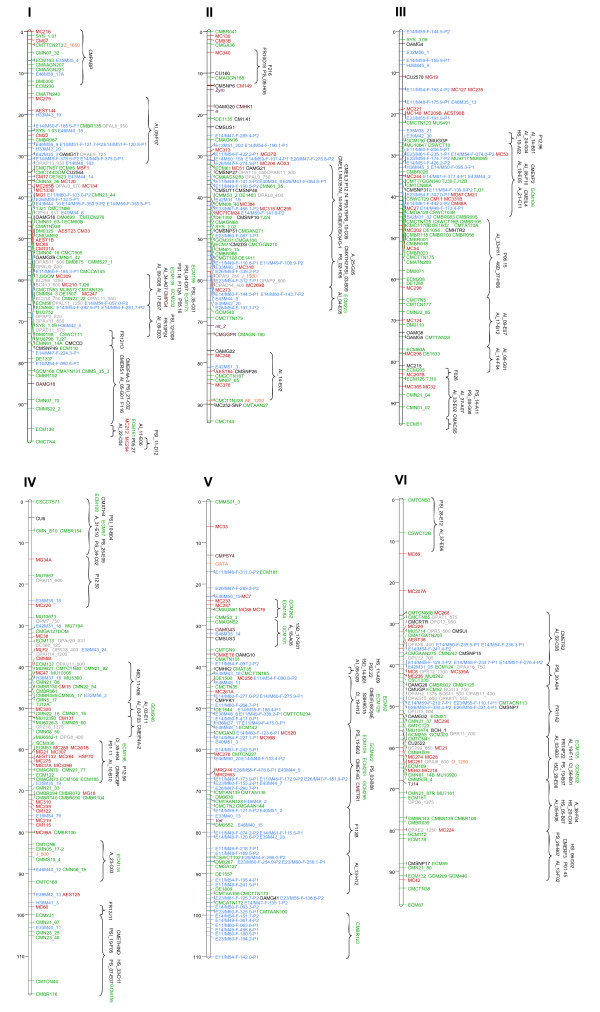
**Integrated melon marker map**. Linkage groups I to VI. Six out of the 12 melon linkage groups (LG) are designated with Roman numerals (I-VI) according to Perin et al. (2002) [11]. Marker type is indicated by colours: SSRs (green), SNPs (black), AFLPs (blue), RFLPs (red), RAPD (grey), IMA (orange), morphological traits (purple) and indel (brown). The map distance is given in centiMorgans (cM) from the top of each LG on the left.

**Figure 2:**
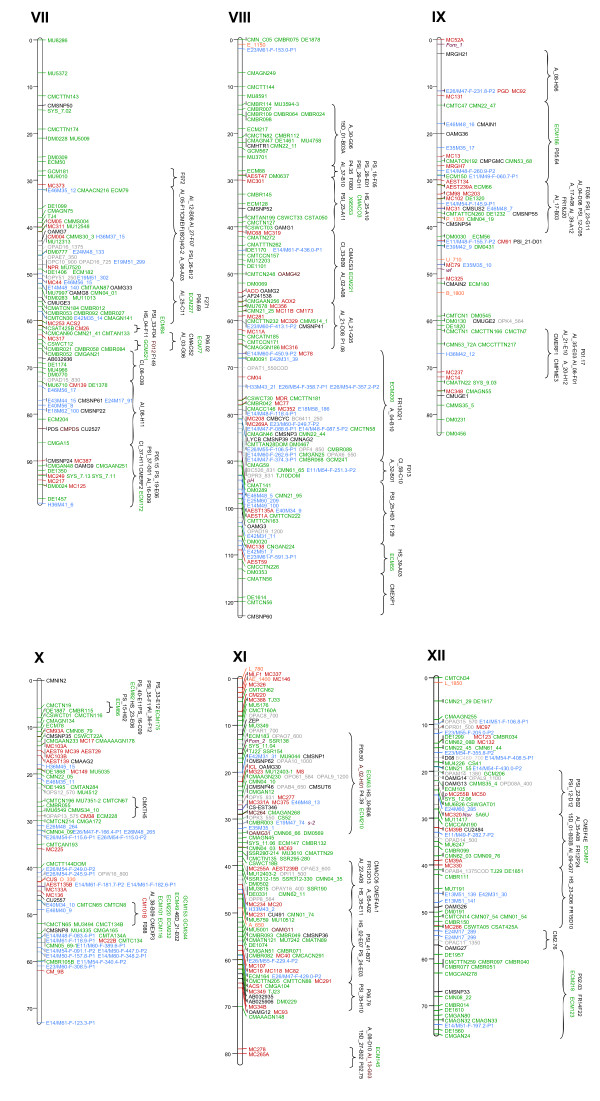
**Integrated melon marker map**. Linkage groups VII to XII. The remaining six linkage groups of melon (VII-XII). Color code for markers are the same as Figure 1.

The framework integrated map contained 1307 markers (110 SNPs, 588 SSRs, 252 AFLPs, 236 RFLPs, 89 RAPDs, 6 indels, 15 IMAs, and 11 morphological traits) spanning 1150 cM that were distributed across 12 LGs with a mean genetic distance between adjacent loci of 0.88 cM (Figures 1 and 2, Additional Files 2 and 3). Integrated map length was similar to previously published maps (Table 1). While the largest marker gap was 11 cM (on the distal ends of LG × and LG IV), the remaining gaps were less than 10 cM, and occurred mainly on the distal ends of LGs (Figures 1 and 2). These gaps are likely due to the lack of sufficient common anchor markers in some maps or slight inconsistencies (distance and/or order) among maps.

Bin-mapping subsequently resulted in the addition of 285 markers (225 SNPs, 52 SSRs, 3 RFLPs, and 5 indels) producing the final integrated map containing 1592 markers (640 SSRs, 335 SNPs, 252 AFLPs, 239 RFLPs, 89 RAPDs, 15 IMAs, 11 indels, and 11 morphological traits) with a mean marker density of 0.72 cM/marker (Table 2 Figures 1 and 2, Additional Files 2 and 3, http://www.icugi.org). One hundred and seventy-eight of these markers were developed, released, or mapped for the first time for the ICuGI Consortium. The marker saturation of this integrated map is far greater than previously published maps (Table 1), increasing dramatically the number of easily transferable markers from 200 [17] to 3353 SNPs and from 386 [18] to 640 SSRs. Noteworthy is the fact that 17 previously bin-mapped markers were positioned on the integrated map after being genotyped in several populations. In each case, these markers mapped to their predicted positions inferred by the bin mapping approach (Table 3), demonstrating the suitability of the bin mapping set [15] to quickly map new markers onto the melon reference map.

**Table 2:** Distribution of genetic markers in the melon integrated map

Linkage Group	Framework markers	Bin markers	Total	Genetic length (cM)	Marker density (cM/marker)
I	131	31	162	99	0.61
II	108	18	126	94	0.74
III	105	23	128	95	0.74
IV	104	27	131	119	0.91
V	115	25	140	110	0.79
VI	102	23	125	98	0.78
VII	108	30	138	99	0.72
VIII	147	30	177	123	0.69
IX	74	18	92	84	0.91
X	89	23	112	73	0.65
XI	131	22	153	80	0.52
XII	93	15	108	77	0.71
					
	1307	285	1592	1150	0.72
					

Distribution and density of markers across the 12 linkage groups, specifying the number of markers that were integrated using Joinmap 3.0 (framework) and bin mapping.

**Table 3:** Comparison of marker positions among bin and integrated melon map

Marker	Linkage group	Bin position (cM)	Integrated map position (cM)
ECM58	I	38-56	58
GCM168	I	75-99	82
CMBR105	III	42-65	42
CMBR100	III	42-65	45
GCM336	IV	52-77	59
GCM255	VI	45-68	55
GCM303	VI	45-68	55
ECM132	VI	80-92	91
ECM182	VII	32-60	49
ECM204	VII	73-86	81
ECM217	VIII	30-41	19
ECM128	VIII	30-41	35
GCM241	VIII	67-90	83
ECM78	X	0-14	11
ECM228	X	26-30	29
ECM164	XI	38-59	59
ECM105	XII	20-41	22
			

Several markers previously mapped using the bin mapping strategy [15] were included in the integrated map. The expected interval for position of the markers in centiMorgans (cM) in the integrated map based on the markers defining the bins according to Fernandez-Silva et al. (2008) [15] is shown in the "Bin position" column, while the actual position in the integrated map is given in the "Integrated map position" column.

Marker distribution in the integrated map varied depending on the marker type. For instance, AFLP markers clustered mainly in certain regions of LGs I, II, III, V, VI, VIII, and × (Figures 1 and 2). AFLP clustering has been commonly reported (e.g., in saturated maps of lettuce [2], potato [42] or tomato [43]), and it is usually associated with heterochromatic regions near centromeres. Even though regions showing AFLP clustering are likely indicative of centromeric positions, comprehensive cytogenetic analyses would be necessary to demonstrate this association in melon. In contrast, SSR, SNP and RFLP markers were generally more evenly distributed throughout the genome. Similar conclusions can not be reached about the remaining markers (RAPDs, IMAs, indels and morphological traits) due to their low number. Nevertheless, SSR marker clustering was observed in LGs III, IV, VII, VIII, XI, and XII, involving mainly SSR markers originated from genomic libraries (e.g., CMBR-SSRs [44]), not from ESTs. This result might indicate that those SSRs are located in repetitive DNA regions as centromeres or telomeres. However, such SSR marker clusters did not overlap those of AFLPs, even though these clusters were in the same LG (i.e., LGs III and VIII), suggesting that SSR marker clustering may be due to reasons not associated with centromeric or telomeric regions.

### Integration of QTL information

**Figure 3:**
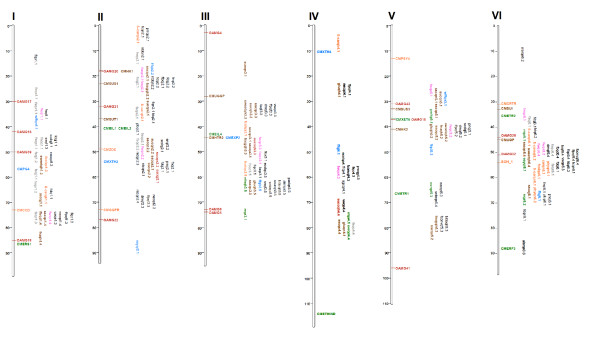
**Quantitative Trait Loci (QTL) positioned in the melon integrated map**. Linkage groups I to VI. QTL are located in a skeleton of the integrated map, where candidate genes for fruit ripening (green), flesh softening (blue), and carotenoid (orange), and sugar (brown) content are also shown. QTL are designated according to additional files 4 and 5 using the same colour code given for the candidate genes.

**Figure 4:**
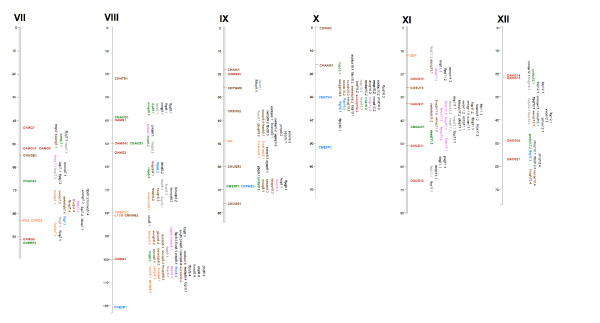
**Quantitative Trait Loci (QTL) positioned in the melon integrated map**. Linkage groups VII to XII. Color codes are indicated in Figure 3.

Eighteen previously reported melon-mapping experiments identified 370 QTL for 62 traits (Table 4 and Additional File 4), and these were aligned in the integrated map described herein. The distribution of these QTL varied from 18 on LG IV to 57 on LG VIII (Figures 3 and 4, Additional File 5). The number of QTLs defined per trait ranged from 1 (e.g., CMV, ETH, and FB) to 40 (FS), with QTL for FS, FW, and SSC being identified in 7, 5, and 5 of the previously reported 18 mapping experiments, respectively. The number of QTL experiments in melon must be considered modest when compared with other major species, with a significant number of the traits being genetically characterized in only one or two different mapping experiments, which thereby limits the meta-analysis of QTL in this species.

**Table 4:** Name and abbreviations of the traits analysed in the current report

Trait	Abbreviation
Ripening rate	RR
Early yield	Eay
Fruit Weight	FW
Fruit Shape	FS
Fruit diameter	FD
Fruit Length	FL
Fruit Convexity	FCONV
Ovary Shape	OVS
Soluble Solid Content	SSC
Fruit number	FN
Fruit Yield	FY
Primary branch number	PB
Percentage of mature fruit	PMF
Flesh firmmes	FF
Seed cell diameter	SCD
Fruit Flesh proportion	FFP
Percent netting	PN
βeta-carotene	β-car, β-carM and β-carE
Ethylene production	ETH
Powdery mildew resistance	PM
Aphis gossypii tolerance	*Ag*
External Color	ECOL
Flesh Color	FCOL
Ring sugar content	RSC
Leaf Area	LA
Total losses	TL
Over ripening	OVR
Finger texture	FT
Water -soaking	WSD
Flesh browing	FB
Fusarium rot	FUS
Stemphylium rot	ST
Fruit flavor	FLV
Necrosis	NEC
Vine weight	VW
Primary root length	PRL
Average diameter of the primary root	PAD
Secondary root density	SRDe
Average lenght of secondary roots	ALSR
Skin netting	SN
Skin thickness	STH
Dry matter	DM
pH	pH
Titratable acidity	TA
3-hydroxy-2,4,4-trimethylpentyl 2-methylpropanoate	PRO
Octanal	OCT
Glucose	GLU
Fructose	FRU
Sucrose	SUC
Total sugars	TSUG
Succinic	SUCC
Sourness	SOUR
Bitterness	BITTE
Sweetness	SWEET
Cucumber mosaic virus	CMV
Net cover	NTC
Net density	NTD
Stripes	STR
Sutures	SUT
Softness	WFF
Total carotenoids	CAR
Phytoene	PHY
α-carotene	αCR
Lutein	LUT
Pentamerous	p
Resistance to Fusarium races 0 and 2	Fom_1
Resistance to Fusarium races 0 and 1	Fom_2
Monoecious	a
Spots on the rind	mt_2
Melon necrotic spot virus	Nsv
Sutures	s-2
Virus aphid transmision	Vat
White flesh	wf
Zucchini Yellow Mosaic Virus	Zym

Even though additional studies would be necessary to draw definitive conclusions, the position of FS QTL tend to be more consistent among experiments than those for FW and SSC QTL, mapping on LG I in six out of seven works, and on LGs II, VI, VII, VIII, XI, and XII in at least three experiments. Clustering of FW and SSC QTL was, however, only observed in LGs VIII and XI, and in LGs II, III, and V, respectively. FS is a highly heritable trait in melon, whereas FW and SSC usually show a lower heritability [25]. The differences in QTL detection among experiments might be partially explained by trait heritability differences. Another possible explanation is that the variability of FS among the germplasm used in the experimental crosses might be controlled by a low number of common QTL with large effects, whereas a higher number of QTL with lower effects and/or more allelic variability among them might be underling SSC and FW.

### Utility of the integrated molecular and QTL map

The integrated map described herein dramatically enhances the development and utility of genomic tools (i.e., markers, map-based cloning and sequencing) over previous melon maps. A large proportion of the markers in the integrated map are SSRs and SNPs, which are easily transferable across laboratories. Moreover, the populations used to construct the integrated map include genotypes from the most important market class cultivars ("Charentais", "Cantaloup", "Hami melon", "Piel de Sapo" and "U. S. Western Shipper") in broad horticultural groups (*cantalupensis, inodorus*, and *reticulatus*), guaranteeing the future utility of the markers in a broad range of cultivars and experimental crosses. The high marker density of the map allows for the selection of specific markers to customize mapping and molecular breeding applications, such as fine mapping, the development of novel genetic stocks (e.g., nearly isogenic lines and inbred backcross lines), MAS, and hybrid seed production.

The positioning of economically important QTL in the integrated map and the standardization of trait nomenclature will facilitate comparative QTL analyses among populations of different origins to provide deeper insights into the genetic control of the diverse phenotypic variability observable in melon germplasm. For example, QTL for SSC on LG III co-localize with QTL associated with SUC, GLU, and SWEET, suggesting perhaps the existence of pleiotropic effects (Figures 3 and 4). The search of candidate genes is also facilitated, as presently little correlation has been detected between candidate gene and trait for ethylene production [45,46], fruit flesh firmness [46], carotenoid content [13,18], or sugar accumulation [18]. These associations were studied in single population, which limits the possibility of identifying associations between candidate genes and QTL. Multi-population analysis is a more powerful approach for detecting QTL/candidate gene associations. For instance, two clusters of QTL involved in carotenoid accumulation and flesh color co-localized with carotenoid-related genes: *CMCRTR *and *BOH_1 *in LG VI and *CMBCYC *and *LYCB *in LG VIII (Figures 3 and 4), and as such become candidate genes for those QTL. Similar associations can been found between genes involved in polysaccharide metabolism and transport and clusters of QTL related to fruit sugar content on LGs II, III, V, VIII, and X. Likewise, associations have been detected between ethylene biosynthesis genes and groups of QTL with effects on fruit ripening on LG VIII.

Preliminary synteny analyses have been conducted between cucumber and melon based on the IRTA SNP and EST-SSR based melon map [17] and the cucumber genome sequence [47]. A large number of EST-based markers (RFLPs, EST-SSRs, and SNPs) mapped in the integrated map will facilitate synteny studies with cucumber and other cucurbit species such as watermelon, squash, and pumpkins as genomic information on such species becomes available. Most cucurbit species display a myriad of variability for economically important vegetative (e. g., branch number, sex expression) and fruit (e.g. morphology, carotenes, sugars) traits. Comparative QTL mapping based on syntenic relationships will allow the evaluation of associations between the allelic constitution at the same genetic loci and the phenotypic variability among the different cucurbit species, as is the case with fruit size between pepper and tomato in Solanaceae family [48].

## Conclusion

Eight molecular marker melon maps were integrated into a single map containing 1592 markers, with a mean marker density of 0.72 cM/marker, increasing dramatically the density over previously published maps in melon. The integrated map contains a large proportion of easily transferable markers (i.e. SSRs and SNPs) and putative candidate genes that control fruit ripening, flesh softening, and sugar and carotenoid accumulation. Moreover, QTL information for 62 traits from 18 different mapping experiments was integrated into the melon map that, together with the mapped candidate genes, may provide a suitable framework for QTL/candidate gene analysis. In summary, the integrated map will be a valuable resource that will prompt the Cucurbitaceae research community for next generation genomic and genetic studies. All the individual maps, the integrated map, marker and QTL information are available at ICuGI web site (http://www.icugi.org). Researchers interested in including their QTL data into the integrated map may contact the corresponding author.

## Methods

### Mapping populations

Eight mapping populations derived from seven independent crosses were used to develop the integrated map (Table 1). Three crosses involved genotypes from the two *C. melo *subspecies (ssp. *melo *and ssp. *agrestis*), three of them between two *C. melo *ssp. *melo *cultivars and one cross between a *C. melo *ssp. *melo *cultivar and a breeding line derived from a cross between *C. melo *ssp. *melo *and *C. melo *ssp. *agrestis *cultivars. The *C. melo *ssp. *melo *genotypes represent the most important economically market classes (Charentais, Cantaloup, Hami melon, Piel de Sapo, and U. S. Western Shipper) belonging to horticultural groups *inodorus, cantalupensis*, and *reticulatus *(Table 1) according to the classification described by Pitrat et al. (2000) [49]. Most of the mapping populations were RILs, where two were F_2 _and one was a double haploid line (DHL) population (Table 1).

Development of new genomic SSR markersNew genomic SSR marker (designated DE- and DM-) were developed by Syngenta seeds. DNA plasmid libraries were constructed using approximately 1 kb fragments of sheared total DNA. SSRs were targeted via 5'-biotinylated total LNA capture probes (12-16 bases long and containing 2, 3, or 4 base repeating units) (Proligo LLC--now IDT). These probes disrupted the double helix of the library DNA at the probe sequence and as a consequence the single strand subsequently formed a double helix with the LNA probe sequence. Streptavidin coated magnetic beads (Invitrogen M-280 Dynabeads) were then used to separate the targeted plasmids from the library. Beads were washed several times and the DNA was then eluted from the beads and transformed into electrocompetent *Escherichia coli *DH12S cells (Life Technologies, California, USA) which were grown up and plated on large Qubit plates. Resultant colonies were then picked using the Qubit, incubated in LB broth, purified and recovered DNA was Sanger sequenced. Proprietary programs selected sequences with SSRs and designed flanking primers.

### Molecular markers

A large proportion of molecular markers developed and/or mapped in previous works (Table 1) were positioned in the integrated map. Additionally, 196 unpublished markers described bellow were included in the merged map. Additional file 2 details the major properties of these markers. On one hand, Syngenta Seeds kindly released 822 SSR markers (see above) to the ICuGI mapping project that were polymorphic in either ARO and/or INRA mapping populations. Eighty-five of them (selected based on their position calculated in in-house built genetic maps by Syngenta, unpublished results) were mapped in the ARO population and subsequently included in the merged map.

On the other hand, new 9 SSRs, 5 indels, s and 27 SNPs were released by ARO group. These indels and SNPs were detected and genotyped according to Harel-Beja et al. (2010) [18] in genes associated with organic acid metabolism or transport (designated OAMG-organic acid melon genes) that were cloned by two methods: (1) from melon cDNA and gDNA by PCR using degenerate primers based on conserved protein sequences; (2) ICuGI database mining. All of them were incorporated to the ARO's map [18].

In contrast, MU- markers are EST-SSRs were developed from their respective EST contigs available at ICuGI web page and mapped by the NERCV group. Four SNPs (AF- and AB- markers) were released by NIVTS (National Institute of Vegetable and Tea Science, Mie, Japan) group and mapped in their respective map. Finally, the unpublished indel MC264 and the SSR marker TJ22 were included in the IRTA map [10,15,17].

### Construction of the integrated map

Various combinations of RFLP, RAPD, IMA, AFLP, SSR, indel and SNP markers had previously been employed to genotyped individuals in each of the eight mapping populations (Table 1). In order to ensure a minimum number of common anchor points among markers, 116 SSR and 1 SNP markers evenly distributed through the melon genome according to two previous linkage maps [15,16] (Additional File 1) were selected to be genotyped in the eight mapping populations. When possible, two markers per anchor-point position were chosen to maximize the probability of identifying polymorphisms in populations examined. Standard, published protocols were employed for SSR marker genotyping [13-16,18].

Marker segregation distortion was investigated employing Joinmap 3.0 software [40] in each of the mapping populations used for map merging. Given the large number of maps and markers evaluated, marker distortion was considered significant at p < 0.005 and when adjacent linked markers also showed distortion at p < 0.01. The heterogeneity of recombination frequency (REC) between common markers among different maps was also evaluated with Joinmap 3.0 and declared significant at p < 0.001.

Initially, a map was constructed for each mapping population, where LGs were defined with the "group" command with a minimum LOD score of 4.0. Groups were then assigned to LGs by comparing their marker composition with the LGs defined in previous reference maps [11,12,15,17]. Groups belonging to the same LG in different populations were then integrated with the "combine groups for map integration" module of Joinmap 3.0 using the following parameters: Kosambi's mapping function LOD > 2, REC < 0.4, goodness of fit jump threshold for removal of loci = 5, performing ripple after adding 1 locus and the third integration round = No. The resulting map was designated the "framework map" and was used in further marker integrations. To add markers mapped by bin mapping [15,17], markers defining the bins in the IRTA map were identified on the framework map. The bins were redefined in the framework map and markers were located subsequently to their respective bins from the IRTA to the framework map..

### Trait and QTL definition

Traits and QTL were selected from 17 published works and 1 unpublished work (Additional Files 4 and 5) by the collaborating project researchers. Crosschecking and evaluation of recording methods allowed for the unification of trait descriptions and common abbreviations were assigned accordingly (Additional File 4). QTL were defined following the directions of the Gramene database [50].

Nevertheless, QTL controlling the same trait expression were often defined in independent publications and/or in different mapping populations and, consequently, QTL characterized in those different populations may correspond to the same genetic locus. Therefore, each QTL was treated independently, making it possible to notice the number of times that a QTL is reported in a similar genomic location across independent experiments.

A specific identifier was assigned to each QTL, where the first letters designate the trait abbreviation, followed by a "Q" that stands for QTL, then a letter indicating a reference to a mapping experiment (publication) followed by a digit representing the LG to which the QTL maps, and then followed by a dot and a final digit that distinguishes different QTL from the same experiment on the same LG (Additional File 5). For example, the designation FDQJ2.2 stands for one of the QTL for FD (fruit diameter) reported in the experiment J and mapping in the LG II.

QTL were defined within a marker interval according to the information presented in the original publication from which it was taken or as a personal communication from a project collaborator. If a flanking marker defining a QTL was not included in the framework map during the merging process, then the next closely linked marker was chosen for representation in the integrated map. Where only a single marker was associated with a QTL, marker position was used as both the start and stop position of the QTL. For illustration purposes, graphic representation of a QTL's position was defined in the centre of a marker interval (Figures 3 and 4).

To provide visual images of their genomic positions, integrated markers and QTL were plotted using Mapchart 2.0 [51]. Colour codes were used to identify marker types, traits, QTL, and candidate genes in order to facilitate visualization of the co-localization of possible QTL and candidate genes involved in similar processes across different mapping experiments.

## Authors' contributions

AJM coordinated the map integration study, provided the marker and QTL data of the IRTA mapping populations, performed the map merging, and drafted the manuscript. MF obtained additional genotype data for the IRTA mapping population. GF integrated QTL information into the merged map, AD assisted in the map merging, prepared tables, and graphic representations and helped to draft the manuscript. PZ and JB formatted the data for representation with C-maps for publication in the ICuGI web site. ZF is the responsible for the ICuGI web site. JES, JZ, and HC provided new marker and QTL data of the USDA-ARS mapping populations; JES assisted with manuscript editing. NF and SM provided new marker and QTL data of the NITVS mapping population and new SNP markers MO provided new marker mapping data for the ARO mapping population and GD developed the DE and DM SSR markers. CD, NB and MP provided new marker and QTL data of the INRA mapping population. RH and PK assisted with map merging construction. RHB, GL, VP, SC, AS, NK, provided new SSR and OGM markers, marker and QTL data of the ARO mapping population. YX and HYZ provided new SSR markers from melon ESTs, and also marker and QTL data of the NERCV mapping population. NF and SM provided the SSR markers used as anchor points for map integration and marker and QTL data of the NITVS mapping population. JGM was the coordinator of the ICuGI project and participated in the design of the study. All authors have read and approved the final manuscript

## Supplementary Material

Additional file 1 **Markers selected as anchor points for map integration**. PowerPoint file depicting a skeleton of the IRTA map [12] and the position of the markers distributed among the collaborating laboratories for use as anchor points for map integration. Click here for file

Additional file 2 **Source of markers**. Excel spreadsheet with two sheets: "Markers in ICuGI consensus map" containing the references in which markers were described and where full details may be obtained, marker type (SSR, Single Sequence Repeat; SNP, Single Nucleotide Polymorphism; RFLP, Restriction Fragment Length Polymorphism; IMA, Inter Microsatellite Amplification; RAPD, Random Amplified Polymorphic DNA; AFLP Amplified Fragment Length Polymorphism; indel, insertion/deletion), the forward, reverse and extension primers (for some SNPs); and "Non-mapped markers" containing the new SSR markers released by Syngenta Seeds that are polymorphic in either ARO and/or INRA mapping populations. Click here for file

Additional file 3 **Integrated melon map**. Excel spreadsheet containing the position of mapped marker on 12 (I-XII) melon linkage groups. Click here for file

Additional file 4 **Consensus vocabulary for the traits positioned on the melon integrated map**. Excel spreadsheet containing consensus definitions for the traits used in the different QTL mapping experiments. Click here for file

Additional file 5 **Quantitative Trait Loci (QTL) located on the melon integrated map**. Excel spread sheet containing the definition of the QTL located on the melon integrated map. QTL are designated according to the following rules: the first letters are the trait abbreviation, followed by a "Q", then a letter indicating the reference followed by a digit representing the LG to where the QTL maps, and the last digit distinguishes different QTL from the same publication in the same LG. The last column indicates molecular markers from the integrated map that flank the mapped QTL. Click here for file
