# Supplementary material for: A consensus linkage map for molecular markers and Quantitative Trait Loci associated with economically important traits in melon (Cucumis melo L.)
Source: BMC Plant Biol. 2011 Jul 28;11:111. doi: 10.1186/1471-2229-11-111 (PMC3163537; doi:10.1186/1471-2229-11-111)
Supplement: Additional file 1 — Markers selected as anchor points for map integration. PowerPoint file depicting a skeleton of the IRTA map [12] and the position of the markers distributed among the collaborating laboratories for use as anchor points for map integration. [file 1471-2229-11-111-S1.PPT]

## Slide 1
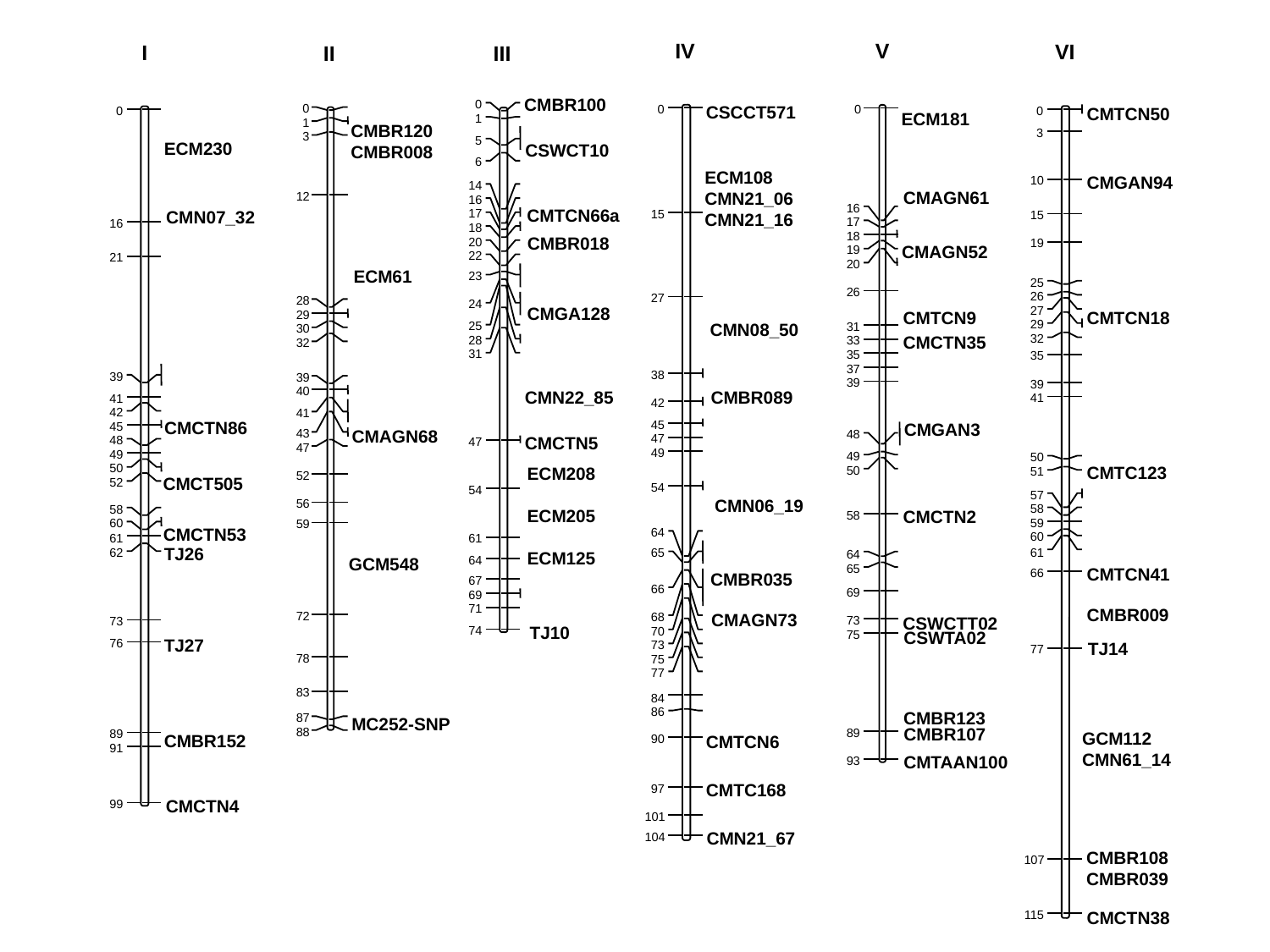

IV
V
VI
I
II
III
CMBR100
0
0
ECM181
0
CSCCT571
0
0
CMTCN50
0
1
1
CMBR120
CMBR008
3
3
ECM230
5
CSWCT10
6
ECM108
CMN21_06
CMN21_16
CMGAN94
10
14
CMAGN61
12
16
CMN07_32
16
CMTCN66a
17
15
15
17
16
18
CMBR018
18
20
19
CMAGN52
19
22
21
20
ECM61
23
25
26
26
27
28
24
CMGA128
27
CMTCN18
CMTCN9
29
CMN08_50
29
25
31
30
32
CMCTN35
28
33
32
31
35
35
37
38
39
39
39
39
CMN22_85
40
CMBR089
41
41
42
42
41
CMCTN86
45
45
CMGAN3
43
CMAGN68
48
47
CMCTN5
48
47
47
49
49
49
50
ECM208
ECM205
ECM125
50
CMTC123
50
51
52
CMCT505
52
54
54
57
CMN06_19
56
58
58
CMCTN2
58
59
60
59
CMCTN53
64
60
61
61
TJ26
61
62
65
64
64
GCM548
65
CMTCN41
66
CMBR035
67
66
69
69
CMBR009
71
72
68
CMAGN73
73
CSWCTT02
73
TJ10
74
70
75
CSWTA02
TJ14
TJ27
76
73
77
78
75
77
83
84
86
MC252-SNP
CMBR123
87
CMBR107
CMBR152
88
89
89
GCM112
CMN61_14
90
CMTCN6
91
CMTAAN100
93
CMTC168
97
CMCTN4
99
101
CMN21_67
104
CMBR108
CMBR039
107
115
CMCTN38

## Slide 2
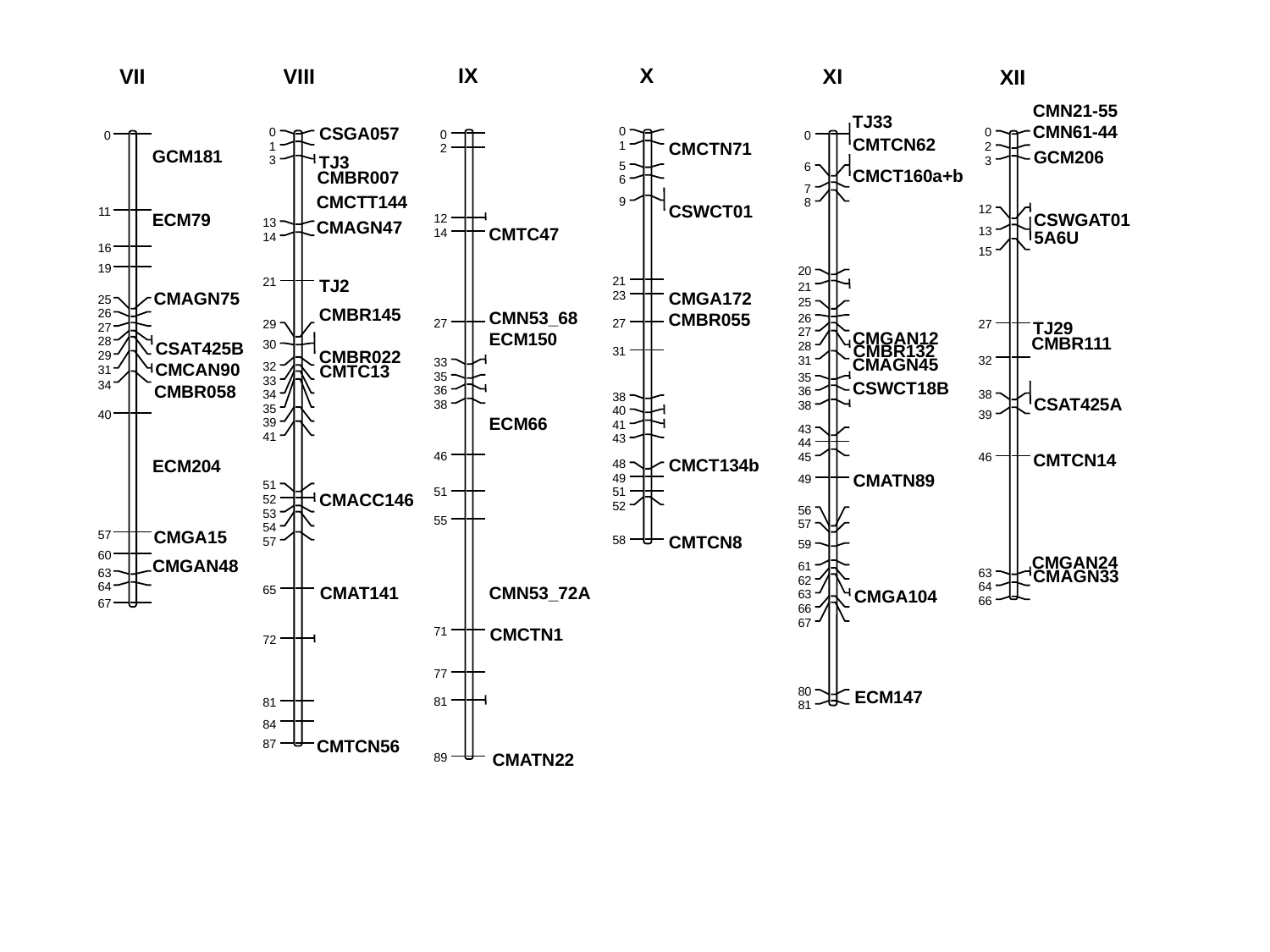

IX
X
VII
VIII
XI
XII
CMN21-55 CMN61-44
TJ33
CSGA057
0
0
0
0
0
0
CMTCN62
1
CMCTN71
1
2
2
GCM181
ECM79
GCM206
TJ3
3
3
5
6
CMCT160a+b
 CMBR007
6
7
CMCTT144
9
8
CSWCT01
12
11
CSWGAT01
CMAGN47
12
13
13
CMTC47
14
5A6U
14
16
15
19
20
21
21
TJ2
21
CMAGN75
23
CMGA172
25
25
CMN53_68
ECM150
ECM66
CMN53_72A
CMBR055
CMBR145
CMBR022
26
26
27
27
29
27
TJ29
27
27
CMBR111
CMGAN12
28
CMBR132
30
CSAT425B
28
31
29
31
32
CMTC13
CMAGN45
33
CMCAN90
32
31
35
35
33
34
CSWCT18B
CMBR058
36
36
34
38
38
CSAT425A
38
38
35
40
40
39
39
41
43
41
43
44
46
ECM204
45
46
CMTCN14
CMCT134b
48
CMATN89
49
49
51
51
51
CMACC146
52
52
56
53
55
57
54
CMGA15
57
CMTCN8
58
57
59
60
CMGAN48
CMGAN24
61
63
63
CMAGN33
62
64
64
65
CMAT141
CMGA104
63
66
67
66
67
71
CMCTN1
72
77
ECM147
80
81
81
81
84
CMTCN56
87
CMATN22
89
